# Long-term effects of sleeve gastrectomy on metabolic parameters and obstructive sleep apnea resolution: a prospective observational study

**DOI:** 10.1007/s11325-026-03570-w

**Published:** 2026-04-17

**Authors:** Sama Abdulrazzaq, Wanis Ibrahim, Abdulaziz Ahmad Al-Hashemi, Aisha Aladab, Shaimaa Sherif Hassan, Tahseen Hamamyh, Mohamed Aly Elsherif, Alyaa Abusabeib, Turki Al-Ahbabi, Maram Saliba, Leyan El-Akhal, Moataz Bashah, Wahiba Elhag

**Affiliations:** 1 Qatar Metabolic Institute, Hamad Medical Corporation, Doha, Qatar; 2 Internal Medicine Department, Hamad Medical Corporation, Doha, Qatar; 3 Pulmonary and Sleep Medicine, Hamad Medical Corporation, Doha, Qatar; 4 Heart Hospital, Hamad Medical Corporation, Doha, Qatar; 5 Weill Cornell Medicine, Al-Rayyan, Qatar; 6 College of Medicine, Qatar University, Doha, Qatar; 7 National Bariatric Center, Surgery Department, Hamad Medical corporation, Doha, Qatar; 8 National Obesity Center, Hamad Medical Corporation, Doha, Qatar

**Keywords:** Sleeve gastrectomy, Obstructive sleep apnea, Apnea hypopnea index, Type 2 diabetes mellitus, Hypertension, Remission

## Abstract

**Background:**

Sleeve gastrectomy (SG) has emerged as the most effective metabolic and bariatric surgical intervention for managing obesity-related complications. The objective of this study is to investigate the long-term effects of SG on cardiometabolic parameters and the resolution of obstructive sleep apnea (OSA) five-year post-surgery.

**Methods:**

This prospective cohort study conducted between (2018–2023) at our institution and included patients aged 18 to 60 years who underwent SG. We compared anthropometric, glycemic, blood pressure (BP), lipid profile and sleep apnea parameters at baseline and 5 years after surgery.

**Results:**

A total of 48 patients included in the study, 70% were female with mean age 35.6 years. Patients achieved significant body mass index reduction from 45.8 ± 8.1 kg/m^2^ at baseline to 30.5 ± 6.82 kg/m² at 5 years with a excess weight loss percentage of 69.14%. Both waist and neck circumferences significantly decreased (*P* = 0.00 for each). Cardiometabolic parameters including blood pressure (BP), fasting blood glucose, hemoglobin A1c (HbA1c), homeostatic model assessment for insulin resistance (HOMA-IR), triglyceride, and high-density lipoprotein (HDL) showed significant improvements (*P* = 0.000 for all). However, changes in total cholesterol and low-density lipoprotein (LDL) did not reach statistical significance. The STOP-BANG questionnaire score decreased from 4.3 at baseline to 1.2 at 5 years. Similarly, the Apnea-Hypopnea Index significantly decreased from 22 at baseline to 6 five years post-surgery. Remission rates were 64.3% for hypertension (HTN), 71.4% for type 2 diabetes(T2DM), 58.1% for dyslipidemia, and 80% for OSA. Approximately 31% of our cohort experienced weight regain; however, they still retained their cardiometabolic improvements and OSA benefits five years after SG.

**Conclusion:**

The current study confirms that SG has a significant long-term impact on both cardiometabolic health with partial or even complete resolution of OSA.

Clinical trial registration: ISRCTN62397779.

## Introduction

Obesity is a chronic relapsing condition with a complex underlying pathogenesis [1]. It is characterized by excessive accumulation of adipose tissue that adversely affects health and elevates the risk of various diseases. It plays a significant role in the development of numerous non-communicable diseases, such as type 2 diabetes (T2DM), hypertension (HTN), dyslipidemia, and obstructive sleep apnea (OSA) [2].Metabolic and bariatric surgery (MBS) is widely recognized as the most effective intervention for achieving sustained weight loss and ameliorating obesity-related comorbidities [3]. In recent years, as the obesity epidemic intensified, MBS evolved to become a standalone surgical specialty, with the annual number of procedures increasing significantly [4]. One of these established bariatric procedures is sleeve gastrectomy (SG), which is currently the most performed MBS worldwide [5]. This is due to its simplicity, safety, effectiveness in promoting weight loss and controlling obesity related complications thereby enhancing cardiometabolic health [6].

Indeed, numerous studies confirmed the short-term efficacy of SG in improving metabolic parameters [7]. However, several aspects remain debatable including weight loss maintenance and the sustained benefits on obesity related complications especially the durability of remission and evolution of cardiometabolic status overtime [8]. Additionally, few studies have assessed the outcomes of SG on OSA despite the fact that OSA is common among patients with obesity undergoing MBS [9]. Furthermore, most of these studies focused on short term outcomes (6–18 months**)** [10]. Except for two long term studies, the first was a retrospective analysis with a small sample size that lacked postoperative polysomnographic data and relied primarily on clinical indicators such as discontinuation of CPAP therapy as surrogate measures of treatment success [11]. In contract, the second study, reported the five years outcome data from two randomized control trials (SLEEVEPASS and SM-BOSS) which evaluated two surgical procedure SG and Roux-en-Y gastric bypass (RYGB) and in these trails the remission of OSA was defined as resolution of symptoms, discontinuation of CPAP therapy, or a reduction in CPAP requirements compared with baseline [12]. Relying solely on the presence or absence of OSA symptoms may be insufficient for accurate assessment, as evidence indicates that the apnea-hypopnea index (AHI) can remain elevated (> 5) even in asymptomatic individuals. Such subclinical OSA can still exert adverse effects on pulmonary function and cardiovascular health, underscoring the importance of objective diagnostic measures [13].

To address this gap, the objective of this prospective cohort study is to evaluate the five-year effects of SG on: (a) changes in anthropometric and cardiometabolic parameters including blood pressure, glycemic control, lipid profile, and sleep apnea indices; (b) remission of obesity-related comorbidities such as hypertension, T2DM, and dyslipidemia; and (c) resolution of OSA as determined by polysomnographic evidence.

## Materials and methods

### Study design and ethics

This is a prospective observational cohort conducted from January 2018 to December 2023 by the Bariatric Medicine, Bariatric surgery and the pulmonary Medicine departments at our institution. It has been approved by ethical committee at the Medical Research Center under the Protocol number 16416/16.

### Participants, procedures, and data collection

The inclusion criteria are adults aged between 18 and 60 years old, who were eligible for MBS, selected SG as their procedure and agreed to participate in the research. Exclusion criteria included previous bariatric procedure (e.g. gastric band, intragastric gastric balloon), current use of steroids, uncontrolled psychiatric illness, alcohol or substance abuse.

Preoperatively, patients were assessed by multidisciplinary teams of specialists including bariatric surgery, bariatric medicine, pulmonary medicine and sleep laboratory technicians. The assessment included detailed history, physical examination and laboratory testing screening as well as sleep study. The patients were initially assessed by bariatric surgeons to determine eligibility, select the appropriate procedure, and obtain informed consent. Then they were assessed by bariatric medicine for medical evaluation, risk stratification and optimization. The STOP BANG questionnaire was administered during this visit, and the score was recorded [14]. Following this, patients were referred to sleep laboratory technicians to be educated on how to use the device. In the current study we utilized Alice PDx home screening polysomnography device to diagnose/screen for OSA. The sleep study results analyzed and documented by sleep laboratory technician and reviewed by pulmonary/sleep physicians. Patients with moderate to severe OSA are referred to the pulmonary sleep clinic, where they received lifestyle and behavioral counselling, including guidance on sleep hygiene and advice to avoid smoking and alcohol. As part of the standard care, Continuous Positive Airway Pressure (CPAP) therapy was offered to all patients with moderate to severe OSA. However, these patients were unable to initiate CPAP prior to SG due to factors such as patient preference, limited device availability, and institutional waiting times. Despite this, they safely proceeded to surgery safely. To ensure optimal care, all patients with moderate to severe OSA who did not receive CPAP were electively admitted to the intensive care unit (ICU) for 24–48 hours postoperatively for close monitoring and observation.

Data collected prior to surgery were demographics [e.g. age, sex], medical comorbidities, type of and number of medications and insulin therapy use, anthropometric parameters [e.g. weight, height, neck circumference, waist circumference (WC)]. Systolic blood pressure (SBP) and diastolic blood pressure (DBP) were also recorded. Laboratory tests included fasting blood sugar (FBS), hemoglobin A1c (HbA1c), insulin level, triglycerides (TG), high-density lipoprotein (HDL), low-density lipoprotein (LDL), total cholesterol (TC)]. Postoperatively, all information were collected again including blood tests and Stop Bang questionnaire. As for polysomnography parameters [apnea hypopnea index (AHI), oxygen desaturation index (ODI)], were obtained only for patients with moderate to severe OSA.

### Definitions of the status and control of comorbidities

Weight regain was defined as regain ≥ 10 kg of the nadir weight reached after SG [15]. To assess diseases status/control post-surgery, We utilized the American Society for Metabolic and Bariatric Surgery (ASMBS) definitions for the classification and reporting of obesity-related complications and metabolic disorders [16]. For OSA screening and diagnosis, the following parameters were measured: AHI which was calculated based on the total numbers of apneas (central, mixed or obstructive) added to the total numbers of hypopneas per hour of sleep [17]. Apnea was defined as a cessation of airflow lasting at least 10 seconds, while hypopnea was characterized by a ≥ 30% reduction in airflow for a minimum of 10 seconds, typically followed by either a ≥ 3% oxygen desaturation or arousal from sleep [17]. The apnea-hypopnea index values were classified as follows: 5–15 was considered mild OSA, 15–30 is moderate, more than 30 is severe OSA. An additional parameter collected and considered crucial in assessing the severity of OSA is the ODI, which quantifies the frequency of oxygen desaturation events per hour of sleep. An event is defined as a drop in oxygen saturation of ≥ 4% from the baseline, persisting for at least 10 seconds. The ODI is calculated by dividing the total number of such events by the total sleep time in hours [18].

### Quality assurance

All physicians who were involved in the research received proper training on patient recruitment, obtaining informed consent, signing the necessary documentation, and explaining the research process to participants, ensuring that the data collected were accurate.

### Statistical analysis

Body mass index (BMI), BMI change, total weight loss percentage (TWL%) and excess weight loss percentage (EWL%) were calculated based on the formula used in previous studies [16]. Insulin resistance was assessed by the homeostasis model assessment of insulin resistance (HOMA-IR) [19]. The student’s t-test for paired samples was used to identify significant differences in the metabolic and sleep-related parameters preoperatively and postoperatively when data were normally distributed. The Wilcoxon signed-rank test was used when the data were not normally distributed. The Pearson correlation test was used to study the relationship between two variables. A two-sided P value of < 0.05 was considered statistically significant. The associated 95% confidence interval was also presented. The post hoc statistical power of the study was calculated using the G*Power software to be 0.69. An observed effect size of 0.30, a sample size of 48, and a significance level of 0.05 were used. All other statistical analyses were performed using SPSS version 23.0 (SPSS, Chicago, IL).

### Surgical technique

All patients underwent laparoscopic sleeve gastrectomy using the same technique, which includes: the division of the gastro-splenic ligament along the greater curvature around 4 cm from the pylorus part of the stomach up to the left diaphragmatic curse with ultrasonic shears. Afterward, the stomach was mobilized and divided along the lesser curvature from the antrum up to the angle of His using buttressed (SeamGuard) linear 60-mm stapler (Covidien Tristapler) or using Echelon Flex over the calibration tube (Midsleeve 38 Fr) introduced into the stomach. The sleeved part of the stomach then removed through the umbilical port. The procedure was concluded with methylene blue leak test from the pylorus part.

## Results

**Fig. 1:**
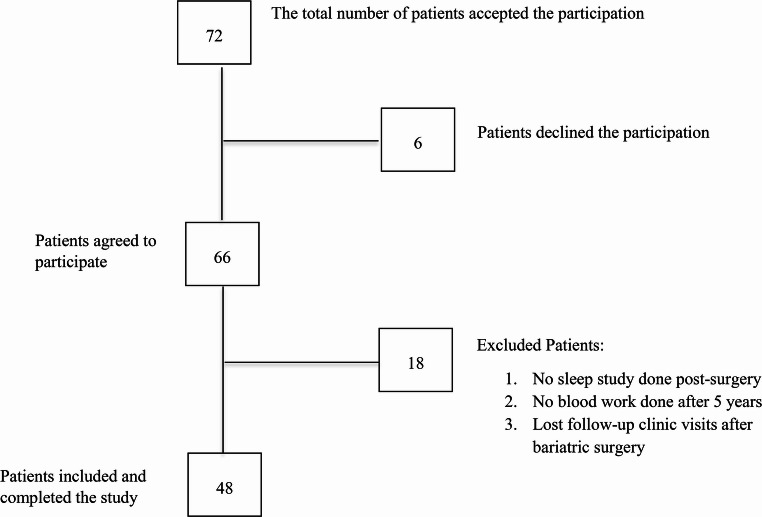
Flow diagram demonstrating number of patients included and excluded in the study

A total of 72 patients were initially invited to participate in the study; However, 6 participants had suboptimal sleep study results due to intolerance and subsequently withdrew from the study after refusing to repeat the test. The remaining 66 patients performed the sleep study as outlined. Eighteen patients were excluded from the analysis for various reasons, including failure to attend the sleep study or blood tests, as well as non-attendance of follow-up visits post-surgery. The remaining 48 patients completed the study period and were included in the final analysis (Fig. 1).

Table 1 presents the baseline characteristics of the study group; the mean age of participants was 35.6 ± 11.4 years with the majority being females (70.8%). Dyslipidemia was the most prevalent comorbidity, affecting 68.75% of patients, with 51.51% of these individuals on medication. This was followed by T2DM, which was present in 33.3% of patients, all of whom were on oral hypoglycemic agents, with 18.75% additionally using insulin therapy. Hypertension was observed in 31.3% of the sample, with 73.3% of the patients on antihypertensive medications. Other comorbidities included hypothyroidism (18.8%) and asthma (14.6%). Sleep study assessments at baseline revealed that 21 patients (43.75%) had normal sleep study, 13 patients (48.14%) had mild OSA, while 14 patients (51.85%) had moderate to severe OSA. Approximately 12% of the patients were smokers.

**Table 1:** Baseline characteristics of the study group (*N* = 48)

Characteristics	Value
Age years (M ± SD)	35.6 ± 11.4
Sex, *n* (%)	
Female	34 (70.8)
Male	14 (29.2)
Comorbidities *n* (%)	
HTN	15 (31.3)
Patients on medications *n* (%)	11(73.3)
T2DM	16 (33.3)
Oral medications *n* (%)	16 (100)
Oral medications + Insulin use *n* (%)	3 (18.75)
Dyslipidemia	33 (68.75)
Patients on medications *n* (%)	17 (51.51)
OSA	27 (56.25)
Mild	13 (48.14)
Moderate to severe	14 (51.85)
Asthma	7 (14.6)
Hypothyroidism	9 (18.8)
Smoking history	6 (12.5)

*M* mean, *SD* standard deviation, *n* number, *T2DM* type 2 diabetes mellitus, *HTN* hypertension

Table 2 displays the changes in anthropometric and weight parameters for five years following SG. A significant weight reduction was observed, with mean weight decreasing from 123.9 ± 30.89 kg to 86.97 ± 22.5 kg. This was accompanied by a substantial decrease in BMI from 45.8 ± 8.1 to 30.5 ± 6.82 kg/m², reflecting a mean change of 15.38 ± 5.9 kg/m². The TWL% and EWL% at five years post-surgery were 33.42% and 69.14%, respectively. Approximately 31.25% of the patients experienced weight regain five years after SG.

**Table 2:** Changes in weight parameters five years after sleeve gastrectomy (*N* = 48)

Anthropometric	Baseline	5 years	*P*
Weight (kg)	123.9 ± 30.89	86.97 ± 22.5	*0.000*
BMI (kg/m²)	45.8 ± 8.1	30.5 ± 6.82	*0.000*
BMI change (kg/m²)	—	15.3 ± 1.28	—
EWL (%)		69.14 ± 20.3	
TWL (%)	—	33.42 ± 9.9	—
WR ≥ 10 kg n (%)	—	15 (31.25)	—

Cell values are mean ± standard deviation, *BMI* body mass index, *EWL%* excess weight loss percentage, *TWL%* total weight loss percentage, *WR* weight regain, *n* number, — not applicable, italics indicate statistical significance

**Fig. 2:**
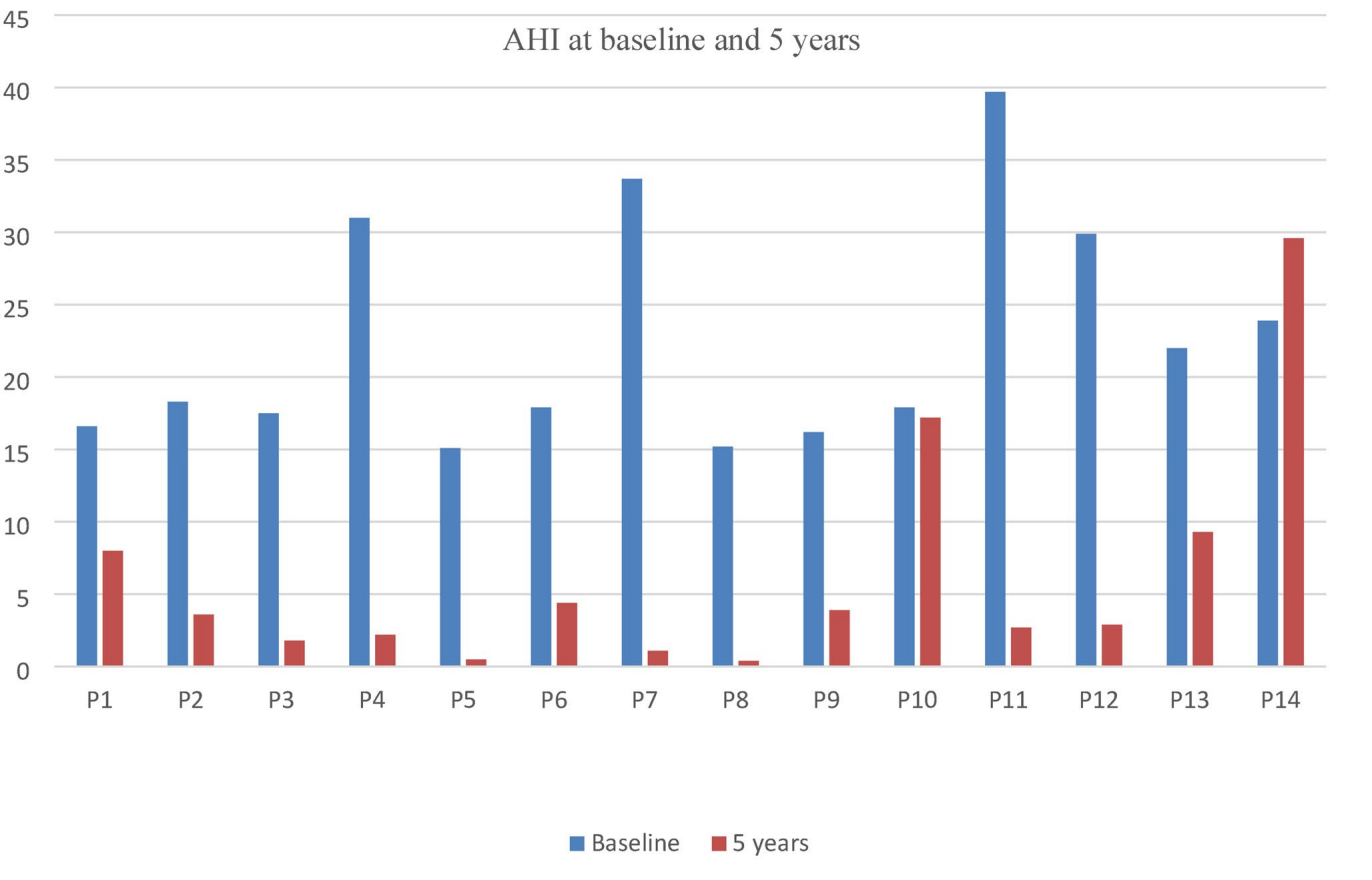
Changes in apnea hypopnea index at baseline and five years after SG for patients with moderate to severe obstructive sleep apnea. *AHI* apnea hypopnea index, *P* patient

Table 3 Compares cardiometabolic and sleep-related parameters at baseline and five years after SG. A significant reduction in neck and waist circumferences was observed (*P* < 0.001 for each). Similarly, a significant reduction in both SBP and DBP was observed following surgery (*P* < 0.001 for each). In terms of lipid profile, there was a significant decrease in TG levels, accompanied by a marked improvement in HDL concentrations (*P* < 0.000 for both). Although TC and LDL levels also showed improvement, these changes did not reach statistical significance. Glycemic parameters also showed significant improvement with the FBS decreasing from 6.47 to 5.00 mmol/L and HbA1c from 5.97 to 5.33% five years after SG (*P* < 0.0001 for each). Markers of insulin resistance also demonstrated significant improvement as evident by reduction in fasting insulin levels accompanied by significant reduction in HOMA-IR (*P* = 0.00 for each). Regarding OSA-related parameters, the STOP-BANG questionnaire scores showed significant improvement, decreasing from a mean of 4.3 preoperatively to 1.2 postoperatively (*P* < 0.000). Similarly, the repeated AHI measurements for patients with moderate to severe OSA showed a significant decline, decreasing from 22 at baseline to five years after surgery. The changes in AHI before and after SG for the 14 patients with moderate to severe OSA who underwent repeat polysomnography at five years are shown in Fig. 2.The desaturation index among this group also demonstrated a substantial improvement, dropping from 18.8 at baseline to 6.2 five years following SG.

**Table 3:** Comparison of cardiometabolic and sleep-related parameters at baseline and five years post sleeve gastrectomy (*N* = 48)

					95% CI of the difference		
Parameter	*N*	Baseline	5 years	Mean difference	Lower	Upper	*P*
Neck circumference (cm)							
Total sample	47	40.9 ± 4.4	33.0 ± 4.9	7.9 ± 41.9	6.63	9.1	*< 0.001*
Male	13	45.4 ± 3.57	37.6 ± 3.16	7.8 ± 2.61	6.22	9.39	*< 0.001*
Females	34	39.2 ± 3.56	31.3 ± 4.40	7.9 ± 4.70	6.24	9.51	*< 0.001*
Waist circumference (cm)							
Total sample	48	134.5 ± 15.87	99.1 ± 21.97	35.4 ± 18.44	30.05	40.77	*< 0.001*
Males	13	143.6 ± 14.10	112.5 ± 14.27	31.1 ± 12.53	23.58	38.72	*< 0.001*
Females	35	131.2 ± 15.52	96.9 ± 15.50	34.3 ± 12.61	29.91	38.72	*< 0.001*
SBP (mmHg)	48	133.5 ± 12.51	117.7 ± 10.38	15.8 ± 13.02	12.05	19.61	*< 0.001*
DBP (mmHg)	48	80.3 ± 11.23	73.2 ± 8.69	7.1 ± 12.43	3.54	10.76	*< 0.001*
FBS (mmol/L)	48	6.47 ± 1.60	5.00 ± 0.62	1.47 ± 1.46	0.01	2.93	*< 0.001*
HbA1c (%)	48	5.97 ± 0.86	5.33 ± 0.58	0.64 ± 0.68	0.44	0.84	*< 0.001*
TC (mmol/L)	48	4.67 ± 0.82	4.46 ± 1.01	0.21 ± 1.02	− 0.088	0.51	0.165
LDL (mmol/L)	48	2.72 ± 0.76	2.60 ± 0.81	0.12 ± 0.75	− 0.103	0.336	0.290
TG (mmol/L)	48	1.67 ± 1.05	0.95 ± 0.44	0.72 ± 0.95	0.445	0.997	*< 0.001*
HDL (mmol/L)	48	1.24 ± 0.28	1.51 ± 0.42	− 0.27 ± 0.37	− 0.380	− 0.164	*< 0.001*
Insulin level(u/dl)	48	32.0 ± 39.2	10.3 ± 5.7	21.7 ± 38.4	10.55	32.85	*< 0.001*
HOMA-IR	48	7.89 ± 5.37	2.2 ± 1.61	5.62 ± 4.81	4.23	7.02	*< 0.001*
STOP-BANG	48	4.3 ± 1.72	1.2 ± 0.68	3.1 ± 1.71	2.57	3.57	*< 0.001*
AHI*	14	22 ± 7.88	6 ± 7.80	16 ± 11.40	9.6	22.2	*0.001*
ODI*	14	18.8 ± 10.56	6.2 ± 5.43	12.6 ± 10.17	3.6	21.5	*0.011*

*N* number, *CI* confident interval, *SBP* systolic blood pressure, *DBP* diastolic blood pressure, *FBS* fasting blood sugar, *HbA1c* hemoglobin *A1c*, *TC* total cholesterol, *LDL* low density lipoprotein lipase, *HDL* high density lipoprotein, *TG* triglyceride, *HOMA-IR* Homeostatic Model Assessment for Insulin Resistance, *AHI* apnea hypopnea index, *ODI* oxygen desaturation index, * test was only repeated for patients with moderate to severe OSA. normal range for neck circumference for male less than 37 cm and female less than 34 cm

Italics indicate statistical significance

Table 4 demonstrates the cardiometabolic and obstructive sleep apnea status five years following sleeve gastrectomy. Approximately, 66% of patients with HTN achieved complete remission, with 33.33% demonstrating improvement. In the cohort with dyslipidemia, 60.60% experienced remission, while 33.33% showed improvement. A small proportion of patients (3.03%) exhibited worsening, and another 3.03% showed no change. Regarding T2DM, half of the patients achieved complete remission, 31.25% experienced partial remission, and 18.75% showed improvement. With respect to OSA, 71.42% of patients attained remission, 21.43% showed improvement and 1 patients experienced worsening OSA.

**Table 4:** Cardiometabolic and obstructive sleep apnea status at five years after sleeve gastrectomy

Disease status	*N* (%)
HTN	
Remission	10 (66.66)
Improvement	5 (33.33)
Dyslipidemia	
Remission	20 (60.60)
Improvement	11(33.33)
Worsening	1(3.03)
No change	1(3.03)
T2DM	
Complete Remission	8 (50)
Partial remission	5(31.25)
Improvement	3 (18.75)
Moderate to severe OSA	14 (51.85)
Remission	10 (71.42)
Improved	3 (21.42)
Worse	1 (7.14)

*N* number, % percentage, *HTN* hypertension, *T2DM* type 2 diabetes mellitus, *OSA* obstructive sleep apnea

Table 5 illustrates the baseline characteristics of the weight regain subgroup. The mean age was 35.6 ± 10.5 years, and the vast majority were female (66.7%). Dyslipidemia was the most prevalent condition in the sample (60%), followed by T2DM (33.3%). Hypertension and hypothyroidism had the same prevalence, each affecting 26.7% of the participants, while asthma had the lowest percentage (20%). OSA was reported in 9 patients (60%), from which 5(55.55%) were mild and 4 patients (44.44%) diagnosed with moderate to severe OSA. The remaining six patients had normal polysomnography findings.

**Table 5:** Baseline characteristics of the subgroup of patients with weight regain post sleeve gastrectomy (*N* = 15)

Characteristic			Value
Age (Years)	Mean ± SD		36.1 ± 10.5
	Median (IQR)		34.0 (28–47)
Sex, *n* (%)			
Male			5 (33.3)
Female			10 (66.7)
Comorbidities n (%)			
Type 2 DM			5 (33.3)
Hypertension			4 (26.7)
Dyslipidemia			9 (60.0)
OSA			9 (60)
Mild			4 (44.44)
Moderate to sever			5 (55.55)
Asthma			3 (20.0)
Hypothyroidism			4 (26.7)
Smoking history			2 (13.3)
Preoperative assessment (M ± SD)			
BMI (kg/m²)			47.8 ± 9.2
Neck circumference (cm)			41.4 ± 4.7
Waist circumference (cm)			133.5 ± 16.0
SBP (mmHg)			132.0 ± 12.6
DBP (mmHg)			83.7 ± 8.0
TC (mmol/l)			4.56 ± 0.92
LDL (mmol/l)			2.76 ± 0.92
TG (mmol/l)			1.41 ± 0.64
HbA1c (%)			5.95 ± 0.94
Insulin level (u/dl)			22.3 ± 8.5
Scale scores (M ± SD)			
HOMA-IR			6.7 ± 3.8
STOP-BANG			4.8 ± 1.9
AHI			12.4 ± 8.9
ODI			10.4 ± 10.1
Weight regains after surgery			
Mean ± SD			16.75 ± 7.7
Median (IQR)			14 (12–18)

*N* number, *SBP* systolic blood pressure, *DBP* diastolic blood pressure, *FBS* fasting blood sugar, *HbA1c* hemoglobin *A1c*, *TC* total cholesterol, *LDL* low density lipoprotein lipase, *HDL* high density lipoprotein, *TG* triglyceride, *AHI* apnea hypopnea index, *ODI* oxygen desaturation index, *IQR* Interquartile Range, normal range for neck circumference for male less than 37 cm and female less than 34 cm

Italics indicate statistical significance

Table 6 presents the cardiometabolic and polysomnographic changes in the subgroup of patients who experienced weight regain after SG. Significant improvements were observed in most anthropometric measures. BMI decreased markedly from 47.9 ± 9.24 to 30.37 ± 8.24, accompanied by reductions in both waist and neck circumferences (*P* = 0.001). Cardiometabolic parameters, including total cholesterol, triglycerides, and HDL, also showed significant improvements compared with pre-SG values, whereas LDL did not change significantly (*P* = 0.851).Regarding glycemic control, significant reductions were noted in FBS, HbA1c, insulin levels, and HOMA-IR. Sleep apnea parameters similarly improved, with the mean STOP-BANG score decreasing from 4.79 ± 1.88 preoperatively to 1.14 ± 0.66 post-surgery (*P* = 0.001). Polysomnography was repeated only for patients with moderate to severe OSA. The mean AHI decreased from 22 ± 7.7 pre-surgery to 2.8 ± 1.57 post-surgery (*P* = 0.043), with all five patients achieving post-surgery AHI values below 5 (normal). The desaturation index also improved from 19.0 ± 12.9 to 7.8 ± 11.4 post-surgery, although this change was not statistically significant (*P* = 0.18).

**Table 6:** Comparison of cardiometabolic and sleep-related parameters at baseline and five years in patients with weight regain after sleeve gastrectomy (*N* = 15)

Parameter	*N*	Baseline	Mean difference	Mean difference	95% CI of the difference		*P* value
					Lower	Upper	
Weight *(kg)*	15	132.87 ± 28.8	95.4 ± 17.0	37.47 ± 29.4	21.18	53.7	*0.001*
BMI	15	47.9 ± 9.24	30.37 ± 8.24	17.42 ± 7.24	13.39	21.46	*0.001*
Neck circumference (cm)	15	41.3 ± 4.9	33.7 ± 3.6	7.6 ± 2.7	6.08	9.20	*0.001*
Waist circumference *(*cm*)*	15	133.5 ± 16	997.5 ± 32	36.0 ± 29.0	19.89	52.10	*0.001*
SBP	15	132.0 ± 12.6	118.5 ± 13.8	13.6 ± 15.5	5.0	22.0	*0.007*
DBP	15	83.73 ± 8.05	72.87 ± 9.80	10.9 ± 7.9	6.9	15.25	*0.001*
FBS	15	6.38 ± 1.73	4.86 ± 0.69	1.52 ± 1.57	0.65	2.39	*0.002*
HbA1c%	15	5.95 ± 0.94	5.16 ± 0.48	0.79 ± 0.71	0.39	1.19	*0.002*
TC (mmol/l)	15	4.56 ± 0.92	4.62 ± 0.72	−0.06 ± 0.59	−0.39	0.25	*0.001*
LDL (mmol/l)	15	2.76 ± 0.92	2.67 ± 0.68	0.89 ± 0.71	−0.30	0.48	0.851
TG (mmol/l)	15	1.41 ± 0.63	1.01 ± 0.45	0.39 ± 0.60	0.06	0.73	*0.009*
HDL (mmol/l)	15	1.21 ± 0.0.25	1.59 ± 0.30	−0.38 ± 0.217	−0.50	−0.26	*0.001*
Insulin level	15	22.8 ± 8.5	8.8 ± 4.3	14.0 ± 7.1	10.0	17.97	*0.001*
HOMA-IR	15	6.72 ± 3.76	1.99 ± 1.28	4.73 ± 3.0	3.0	6.4	*0.001*
STOP-BANG	15	4.79 ± 1.88	1.14 ± 0.66	3.64 ± 2.09	3.0	6.4	*0.001*
AHI*	5	22.0 ± 7.7	2.8 ± 1.57	19.3 ± 7.9	9.4	29.1	*0.043*
ODI	5	19.0 ± 12.9	7.8 ± 11.4	11.3 ± 20.2	−13.8	36.4	0.180

*N* number, *CI* confident interval, *BMI* body mass index, *SBP* systolic blood pressure, *DBP* diastolic blood pressure, *FBS* fasting blood sugar, *HbA1c* hemoglobin *A1c*, *TC* total cholesterol, *LDL* low density lipoprotein lipase, *HDL* high density lipoprotein, *TG* triglyceride, *HOMA-IR* Homeostatic Model Assessment for Insulin Resistance, *AHI* apnea hypopnea index, *ODI* oxygen desaturation index, * test was only repeated for patients with moderate to severe OSA. normal range for neck circumference for male less than 37 cm and female less than 34 cm. Italics indicate statistical significance

## Discussion

Obesity is a major risk factor for T2DM, HTN, dyslipidemia and OSA. While various medical treatments exist for these conditions, surgical approach such as SG have proven to be superior in achieving remission of obesity associated complication [20]. This prospective cohort study is unique in providing comprehensive data on long term outcomes of SG, assessing several cardiometabolic parameter and offering in-depth evaluation of OSA, a common respiratory disorder among patients with obesity using both subjective and objective measures. The main findings of the current study is that SG resulted in significant long term weight loss as well as substantial improvement in blood pressure, glycemic parameters, lipid profile and sleep apnea parameters. This was accompanied by remission in multiple cardiometabolic complications related to obesity including HTN, T2DM, dyslipidemia and OSA. Collectively these findings confirm the durability of benefits achieved through BMS such as SG.

In terms of anthropometric outcomes, the present study demonstrated significant reductions in weight and BMI, with a mean BMI change of 15.38 kg/m² and a TWL% of 33.45 ± 9.9%. These findings are consistent with prior research reporting a BMI reduction of 33.2 ± 6.2 kg/m² and a TWL% of 28.1% five years following SG [21]. The observed weight loss is primarily due to the restrictive effect of SG, further enhanced by significant hormonal and metabolic changes. These include a reduction in circulating ghrelin levels and an increase in incretin response specifically, elevated secretion of peptide YY and glucagon-like peptide-1 (GLP-1) which together promote early satiety and decreased caloric intake [22]. In addition to the weight loss observed after SG, a significant reduction in WC was also noted, consistent with previous research reporting a 31% decrease WC following SG suggesting potential improvements in cardiovascular risk and insulin resistance [23]. Moreover, WC has been identified as a strong predictor of metabolic success after SG. For instance, one study reported a significant inverse correlation between WC and EWL%, with each one cm reduction in WC associated with a 12% increase in the likelihood of achieving favorable metabolic outcomes [24].

One of the outcomes assessed in this study was long-term weight regain. In our cohort, 31.25% of patients experienced weight regain five years after SG. Weight regain is a well-documented phenomenon following MBS and continues to pose a significant clinical challenge. However, accurately estimating its prevalence remains difficult due to the lack of a universally accepted definition of weight regain [15]. Previous research demonstrated that long term weight regain ranging between 29% and 76% [25]. Weight regain after bariatric surgery is multifactorial in nature, influenced by alterations in gut hormones, metabolic adaptations, dietary non-adherence, psychological factors, maladaptive eating behaviors, sedentary lifestyles, as well as anatomical or surgical factors [15]. Effective management requires a multidisciplinary approach involving dietary therapy, psychological support, behavioral counseling, and structured exercise interventions, along with close follow-up and ongoing monitoring by a dedicated bariatric team [15].

In terms of cardiometabolic parameters, both systolic and diastolic blood pressure considerably decreased in the current sample after SG, with 64.28% of patients achieving hypertension remission and 35.71% showing improvement. These findings are in line with previous research, which reported a 41.4% complete resolution and 24.3% partial resolution of hypertension five years post-SG, outcomes comparable to those observed with gastric bypass [26]. Similarly systematic review found that hypertension resolved in 62.17% of cases and improved in 35.7% 5 years after surgery [27]. Hypertension remission following SG may be attributed to reduced insulin resistance, decreased inflammation, and lower activity of the Renin-Angiotensin System, resulting in less arterial stiffness and sodium reabsorption [27].

In terms of glycemic parameters, we observed significant improvements following SG. There were significant reductions in FBS, HbA1c, and markers of insulin resistance including fasting insulin levels and a HOMA-IR consistent with findings reported from previous studies [20]. Approximately 71% of our patients achieved remission of T2DM, while 28.57% showed notable improvement. Other studies have reported complete remission rates ranging from 9% to 78% at five years post-SG [28], with improvement rates varying between 10% and 52% over the same period [28]. This wide variability is largely attributed to differences in the criteria used to define T2DM remission, as well as patient-specific factors such as diabetes duration, baseline glycemic control, and disease severity [29].The observed improvements in T2DM following SG are likely driven by multiple mechanisms, including altered neurohormonal regulation, increased secretion of GLP-1, and favorable shifts in gut microbiota composition [22].

Regarding the lipid profile, the current study demonstrated remarkable reduction in lipid parameters, particularly in TG and HDL levels with remission rate of dyslipidemia reaching 58%. The improvement in lipid profile we observed is consistent with previous reports after SG [30]. Dyslipidemia associated with obesity typically includes hyperglycemia, small, dense LDL particles, and low HDL levels; all reflecting insulin resistance commonly observed in obesity. As insulin sensitivity improves with weight loss, lipid parameters also improve [31].

Obstructive sleep apnea is characterized by repeated upper airway collapse during sleep, leading to reduced or absent respiratory airflow and resulting in sleep fragmentation along with numerous clinical manifestations [32]. OSA is commonly observed in patients with severe obesity, particularly those undergoing bariatric surgery and is often underdiagnosed despite its typical severe presentation [33]. It is estimated that up to 70% of patients evaluated for bariatric surgery present with OSA [34]. In our sample, 50% of patients were diagnosed with moderate to sever OSA. Following SG, these patients demonstrated significant overall improvements in multiple OSA-related parameters. Notably, scores on the STOP-BANG questionnaire improved substantially postoperatively, indicating a reduction in OSA symptoms. These findings are consistent with previous short-term studies that have reported improvements in STOP-BANG scores post-SG [35]. The severity of OSA is typically assessed using AHI. In our study, we observed a substantial reduction in AHI, with the mean decreasing from 22 events per hour at baseline to 6 events per hour at the five-year follow-up. This finding indicates a sustained and clinically meaningful improvement in OSA severity. Similar findings have been reported in the literature. For example, Timmerman et al. (2019) reported a decrease in mean AHI from 45.8 to 11.3 events per hour within six months of surgery [36]. Other studies have also confirmed significant improvements in AHI following SG [37].The desaturation index also showed significant improvement, in line with findings from a previous study reporting reduction in ODI from 51.4 to 21.3 one year post SG [10]. Furthermore, most patients in our study (80%) achieved remission of OSA. This remission rate is comparable to those reported in previous studies, which ranged from 50% to 87% [10]. Notably, most prior studies assessed short term outcomes (6–12 months), while long-term data are limited. Two studies have reported long-term outcomes. One study found a 55% remission rate five years post-SG, based on CPAP/BiPAP discontinuation without repeat polysomnography [11]. Another study reported a 60% remission rate at five years, defined by symptom resolution and reduction or cessation of CPAP use [12]. Only one patient in our cohort experienced worsening of OSA, with AHI increasing from 23.9 preoperatively to 29.6 five years post-operatively. This patient had multiple comorbidities, including T2DM, hypertension, dyslipidemia, uncontrolled hypothyroidism, and ischemic cardiomyopathy, along with an 8-kg weight regain, which may explain the unfavourable response. Medication non-compliance and recurrent fluid overload may have also contributed to this outcome.

A significant factor contributing to the improvement of OSA following SG is the significant reduction in neck circumference. In our study, patients demonstrated a decrease from a mean of 40.9 cm to 33.0 cm, which is consistent with findings from a previous study reporting a reduction from 43.9 cm to 40.2 cm nine months post-SG [36]. Previous research demonstrated that this anatomical change is associated decreased intratissue fat within the tongue, pharyngeal lateral walls, and soft palate [38]. These changes contribute to an increase in velopharyngeal airway volume, ultimately leading to an improvement in OSA symptoms [38]. Additional contributing factors encompass substantial weight loss, hormonal changes such as improved insulin sensitivity and increased in GLP-1 level. Collectively, these factors underscore the complex interplay between adiposity, metabolic regulation, and the pathophysiology of sleep-disordered breathing, highlighting how improvements in metabolic and anthropometric parameters contribute to the amelioration of OSA symptoms [36].

Despite weight regain in our cohort, patients continued to show significant improvements in most cardiometabolic and polysomnographic parameters. Previous research has demonstrated that even among patients with substantial weight regain (> 10 kg), 61% maintained complete remission, 22% had persistent symptoms, and only 17% experienced relapse [37]. These findings support the overall therapeutic benefit of sleeve gastrectomy in morbid obesity, even when some weight is regained during long-term follow-up [37]. To our knowledge, no prior studies have comprehensively assessed other cardiometabolic and polysomnographic outcomes in patients with weight regain, highlighting an important area for future research.

This study has several limitations that should be acknowledged. A larger sample size would have improved the statistical power and generalizability of our findings. Additionally, including detailed documentation of lifestyle factors, such as dietary habits and physical activity, would have allowed a more comprehensive assessment of variables influencing outcomes after SG. Evaluating factors associated with weight regain in the subgroup of patients who experienced it could have helped identify individuals at risk and provided guidance for tailored treatment strategies. Furthermore, complications related to SG, such as immediate postoperative intensive care unit admissions or prolonged hospital stays, were not recorded. Including this data in future research could enrich our understanding of perioperative risk profiles and recovery patterns. Moreover, evaluating OSA related comorbidities such as obesity hypoventilation syndrome, as well as psychological conditions like depression and anxiety would provide a more comprehensive view of the interplay between SG and OSA. Although CPAP therapy was not utilized by all patients with moderate to severe OSA due to logistical constraints and individual patient preferences, this offers a unique strength. The absence of CPAP use eliminates a major confounding variable, allowing for a more isolated evaluation of SG’s direct impact on AHI reduction an effect clearly demonstrated in our results [39]. Despite these limitations, the current study has notable strengths. It is a prospective, long-term study that evaluated the impact of SG on multiple cardiometabolic disorders, including hypertension, T2DM, and dyslipidemia, using the ASMBS criteria [16]. The study also provided a comprehensive evaluation of OSA outcomes, employing both subjective and objective measures. These methodological strengths provide compelling evidence supporting SG as an effective intervention capable of producing sustained improvements in OSA.

## Conclusion

This study contributes to the growing body of evidence supporting the enduring benefits of SG. The findings affirm the long-term efficacy of SG in achieving sustained weight loss and mitigating obesity-related complications. Specifically, the study highlights significant and durable improvements in insulin resistance, type 2 diabetes, hypertension, dyslipidemia, and obstructive sleep apnea, with these positive effects persistently maintained for up to five years post-surgery.

## Data Availability

Data will be made available on reasonable request.
